# Inadequate BiP availability defines endoplasmic reticulum stress

**DOI:** 10.7554/eLife.41168

**Published:** 2019-03-14

**Authors:** Milena Vitale, Anush Bakunts, Andrea Orsi, Federica Lari, Laura Tadè, Alberto Danieli, Claudia Rato, Caterina Valetti, Roberto Sitia, Andrea Raimondi, John C Christianson, Eelco van Anken

**Affiliations:** 1 Division of Genetics and Cell BiologySan Raffaele Scientific InstituteMilanItaly; 2 Università Vita-Salute San RaffaeleMilanItaly; 3 Ludwig Institute for Cancer ResearchUniversity of OxfordOxfordUnited Kingdom; 4 Cambridge Institute for Medical ResearchUniversity of CambridgeCambridgeUnited Kingdom; 5 Department of Experimental MedicineUniversity of GenovaGenovaItaly; 6 Experimental Imaging CenterSan Raffaele Scientific InstituteMilanItaly; Howard Hughes Medical Institute, University of California, San FranciscoUnited States; Howard Hughes Medical Institute, University of California, BerkeleyUnited States

**Keywords:** unfolded protein response, endoplasmic reticulum, proteotoxicity, ER stress, chaperones, BiP/GRP78, Human

## Abstract

How endoplasmic reticulum (ER) stress leads to cytotoxicity is ill-defined. Previously we showed that HeLa cells readjust homeostasis upon proteostatically driven ER stress, triggered by inducible bulk expression of secretory immunoglobulin M heavy chain (μ_s_) thanks to the unfolded protein response (UPR; Bakunts et al., 2017). Here we show that conditions that prevent that an excess of the ER resident chaperone (and UPR target gene) BiP over µ_s_ is restored lead to µ_s_-driven proteotoxicity, i.e. abrogation of HRD1-mediated ER-associated degradation (ERAD), or of the UPR, in particular the ATF6α branch. Such conditions are tolerated instead upon removal of the BiP-sequestering first constant domain (C_H_1) from µ_s_. Thus, our data define proteostatic ER stress to be a specific consequence of inadequate BiP availability, which both the UPR and ERAD redeem.

## Introduction

It is well-established that accumulation of unfolded proteins in the endoplasmic reticulum (ER)—a condition referred to as ER stress—activates the unfolded protein response (UPR), which, in turn, mitigates the stress, most notably through enhancing the ER chaperone content to boost the protein folding capacity (Walter and Ron, 2011). What defines ER stress, and how ER stress may engender cytotoxicity, however, are poorly understood issues. Moreover, it is still debated what feature of ER stress activates the UPR. An important reason why these are still open questions is the wide-spread use of ER stress-eliciting drugs, such as tunicamycin (Tm), which inhibits N-glycosylation, or thapsigargin (Tg), which causes Ca^2+^ efflux from the ER (Walter and Ron, 2011). These drugs have pleiotropic effects and are inherently cytotoxic, hence obscuring important aspects of how ER homeostasis can be restored by virtue of the UPR or not. To overcome the shortcomings of ER stress-eliciting drugs, we recently have developed a HeLa cell-based model for proteostatically driven ER stress (Bakunts et al., 2017). Inducible overexpression of the IgM subunits µ_s_ and the λ light chain, in stoichiometric amounts, leads to bulk secretion of IgM with little if any UPR activation. In the absence of λ, however, µ_s_ is retained in the ER, and maximally activates the three main UPR branches, governed by IRE1α, PERK, and respectively, ATF6α. Yet, the cells successfully adapt to the proteostatic insult by expanding the ER both in size and in chaperone content, such that cell viability and growth are unaffected in the process, and UPR signaling subsides to a submaximal amplitude once homeostasis is restored (Bakunts et al., 2017).

The ER resident chaperone BiP stands out in the course of the adaptation to µ_s_ expression in two ways. First, ER stress sensing and UPR signaling occur in a µ_s_/BiP ratiometric fashion, that is the amplitude of UPR signaling is maximal when µ_s_ levels eclipse those of BiP, which is sequestered through binding to µ_s_, while UPR signaling subsides to submaximal output when an excess of BiP over µ_s_ is restored (Bakunts et al., 2017). ER homeostatic readjustment is due to the UPR, since BiP is a key UPR target gene (Walter and Ron, 2011). Second, ER homeostatic readjustment to µ_s_ expression causes a ∼10-fold increase of BiP levels overall, which entails that BiP shifts from about one tenth to about one third of the total protein mass in the ER, such that BiP is the only chaperone in the ER of which the levels outmatch those of µ_s_ (Bakunts et al., 2017).

The two main models that have been proposed for UPR activation are that it entails i) dissociation of BiP from the lumenal domains of the main ER stress sensors, IRE1α, PERK (Bertolotti et al., 2000) and ATF6α (Shen et al., 2002), and ii) direct binding of unfolded proteins (Gardner and Walter, 2011; Karagöz et al., 2017), including the Ig heavy chain C_H_1 domain (Karagöz et al., 2017), to these sensors. Based on insights obtained from µ_s_-driven ER stress, we argue that these two UPR activation models are not mutually exclusive. Rather, the two models are complementary and should be unified, since in a three-way competition between UPR sensors, BiP, and an ER client protein (µ_s_) for binding one another, the ratio of UPR sensors bound to the client versus those bound to BiP most robustly report on the client/BiP ratio, to which indeed the UPR signaling amplitude correlates (Bakunts et al., 2017).

HeLa cells tolerate genetic ablation of the main three UPR transducers, but expression of µ_s_ in the context of UPR-ablated cells causes synthetic lethality through apoptosis, underscoring the key role the UPR has in restoring ER homeostasis (Bakunts et al., 2017). In this study we exploited this synthetic lethality to define how ER stress becomes proteotoxic.

## Results

### IRE1α and PERK are expendable, but ATF6α is key for µ_s_-provoked ER homeostatic readjustment

**Figure 1.**
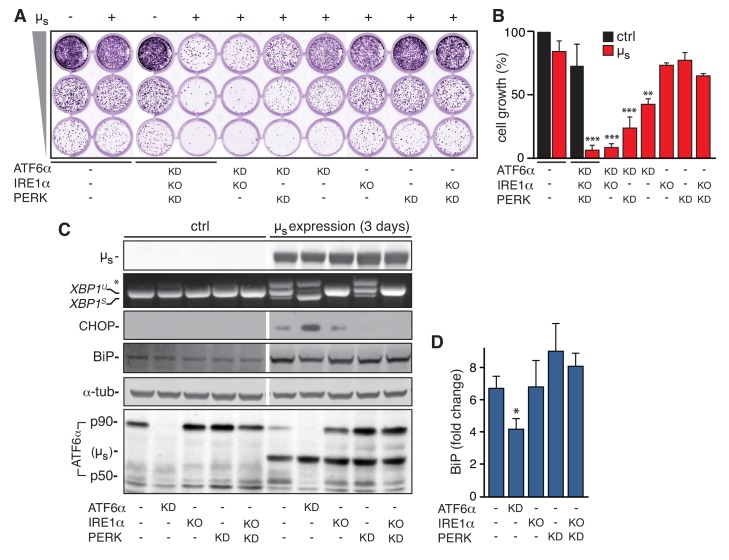
ATF6α is essential but IRE1α and PERK are dispensable for restoring ER homeostasis upon µ_s_ expression. (**A–D**) In HeLa-µ_s_ cells, IRE1α was deleted (KO), and ATF6α and PERK were silenced (KD) either alone or in combination, or not (-), as indicated. (**A**) Cells were seeded upon 1:5 serial dilution into 24-well plates, and treated with 0.5 nM mifepristone (Mif) to induce expression of µ_s_ where indicated (+). After 7 days of growth, cells were fixed and stained with crystal violet. (**B**) Staining in (**A**) was quantitated as a measure for cell growth. Mean and s.e.m. are shown in a bar graph; n = 2. (**C**) Expression of µ_s_ was induced for 0 or 3 days. Immunoblotting of lysates from cells that were sufficiently viable upon the insult for analysis revealed levels of µ_s_, BiP, CHOP, α-tubulin, and ATF6α processing (i.e. release of the p50 cleavage product from the p90 precursor); cross-reaction of the secondary antibody against anti-ATF6α with µ_s_ is denoted (µ_s_). RT-PCR fragments corresponding to spliced (*XBP^S^*) and unspliced (*XBP^U^*) were separated on gel. A hybrid product that is formed during the PCR reaction is denoted by an asterisk. (**D**) BiP levels in (**C**) were quantitated and expressed as fold change upon µ_s_ expression compared to untreated cells. Mean and s.e.m. are shown in a bar graph; n=2-5. Statistical significance of differences in growth (**B**), or in expression levels (**D**), was tested by ANOVA (*p ≤ 0.05; **p ≤ 0.01; ***p ≤ 0.001). 10.7554/eLife.41168.003Figure 1—source data 1.

To investigate in detail how the UPR sustains ER homeostatic readjustment to bulk µ_s_ expression, we exploited cells in which IRE1α was deleted and PERK and ATF6α were silenced with good efficiency (Bakunts et al., 2017), either individually or in combinations. Surprisingly, ablation of IRE1α and PERK (either individually or in combination) had negligible effects on viability and growth of µ_s_-expressing cells, (Figure 1A,B), or on ATF6α activation (Figure 1C). Thus, IRE1α and PERK are dispensable for restoring ER homeostasis upon bulk µ_s_ expression, and ER stress levels are not enhanced in their absence (although there is some ATF6α activation already under basal conditions when IRE1α and PERK are ablated; Figure 1C). Conversely, silencing of ATF6α alone caused reduced growth and/or viability of µ_s_-expressing cells (Figure 1A,B), implying that ER homeostasis was not (fully) restored.

When µ_s_ is expressed for 3 days in wild-type cells, ER homeostasis is restored, and, consequently, IRE1α and PERK signaling subsides to submaximal output (Bakunts et al., 2017). In ATF6α-silenced cells, conversely, ER homeostasis is not restored, and, accordingly, signaling through the PERK and IRE1α pathways remained persistently high (Figure 1C); that is levels of CHOP, a key downstream effector of PERK (Harding et al., 2000), were increased, and IRE1α-mediated XBP1 mRNA splicing (Calfon et al., 2002) was enhanced, as was evident from the increased prominence of the higher mobility band, corresponding to the RT-PCR product of the *XBP1^S^* transcript from which the intron has been removed (Calfon et al., 2002). Ablation of ATF6α in combination with ablation of IRE1α and/or PERK caused apoptosis (Bakunts et al., 2017) and, consequently, abrogated viability of µ_s_-expressing cells (Figure 1A,B). We concluded that accumulation of µ_s_ in the ER per se confers proteotoxicity when the UPR is dysfunctional, and that the UPR counteracts this proteotoxicity, in particular through the ATF6α branch.

### IRE1α and PERK are expendable, but ATF6α is key for ER expansion in response to µ_s_ expression

**Figure 2.**
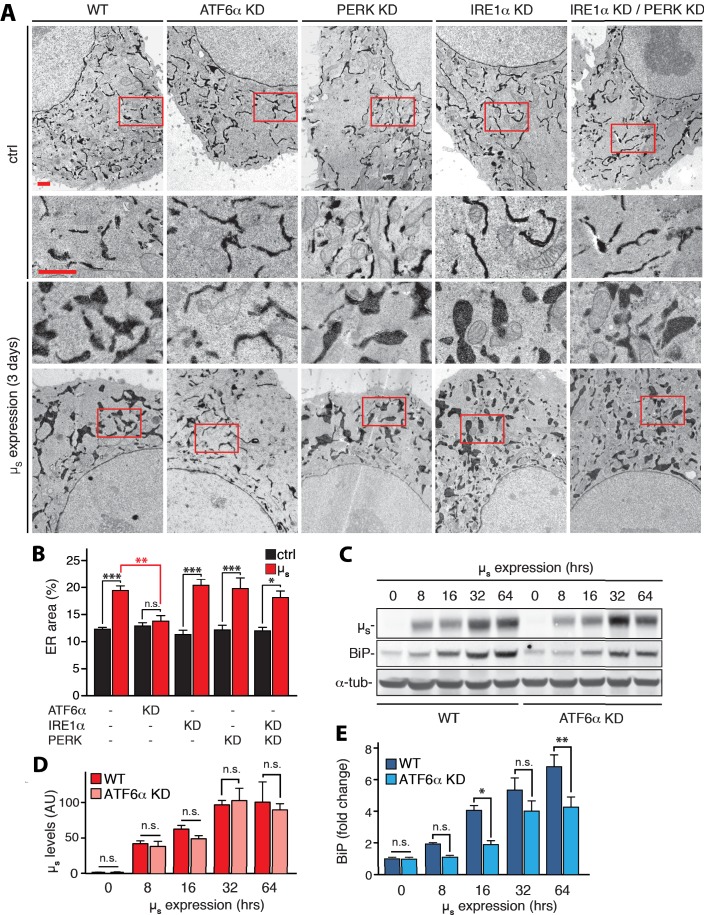
ATF6α is essential but IRE1α and PERK are dispensable for upregulation of ER chaperones and ER expansion in response to µ_s_ expression. (**A,B**) HeLa-µ_s_ cells in which UPR transducers were ablated by silencing alone or in combination, or not (WT), as indicated, were induced with 0.5 nM Mif to express µ_s_ for 3 days or not. The cells harbor APEX-KDEL, a modified version of pea peroxidase that is targeted to the ER, and that catalyzes polymerization of 3,3’-diaminobenzidine tetrahydrochloride (DAB) upon treatment with H_2_O_2_ to obtain DAB precipitates (dark), revealing the extent of the ER in electron micrographs. Boxed areas are shown by 3-fold magnification; scale bars represent 1 µm (**A**). The extent of ER expansion was assessed as described (Bakunts et al., 2017), and the percentage of the area within the cytoplasm corresponding to ER was determined and depicted in bar graphs (**B**). Mean and s.e.m. are shown, n = 10–20. (**C–E**) Cells were induced to express µ_s_ for the indicated times. Levels of µ_s_ (**D**) and BiP (**E**) were quantitated from (**C**), and replicate experiments. (**D**) Levels in WT of µ_s_ at 64 hr were set at 100 that was scaled to levels of BiP in WT at 64 hr such as to reflect a ratio of µ_s_ to BiP of 2:3, that is an estimate for this ratio at day three based on earlier quantitations that we have described (Bakunts et al., 2017). Mean and s.e.m. are shown in bar graphs; n = 2–5. Statistical significance in the extent of ER areas in the electron micrographs between µ_s_-expressing or non-expressing cells (black), or between µ_s_-expressing WT or ATF6α ablated cells (red) (**B**), or in expression levels (**D,E**) was tested by ANOVA (n.s., not significant; *p≤0.05; **p≤0.01; ***p≤0.001). 10.7554/eLife.41168.005Figure 2—source data 1.

Despite the persistently maximal signaling through the PERK and IRE1α pathways upon µ_s_ expression in ATF6α-silenced cells (Figure 1C,D), upregulation of BiP was compromised (Figure 1C,D; Figure 2C,E), while upregulation of two other ER chaperones, PDI, and GRP94 was abolished (Figure 2—figure supplement 1), which confirms that also these ER chaperones are prominent ATF6α targets (Bommiasamy et al., 2009). ATF6α silencing did not affect accumulation of µ_s_ (Figure 2C, D), however, and the ER did not expand (Figure 2A, B), in accordance with the compromised upregulation of ER chaperones. Conversely, ER expansion (Figure 2A, B), and BiP upregulation (Figure 1C, D) upon µ_s_ expression was not compromised in PERK– and/or IRE1α–ablated cells. Thus, the ATF6α branch of the UPR is the main if not sole driver of ER expansion in response to µ_s_ expression. 

**Figure 2—figure supplement 1.**
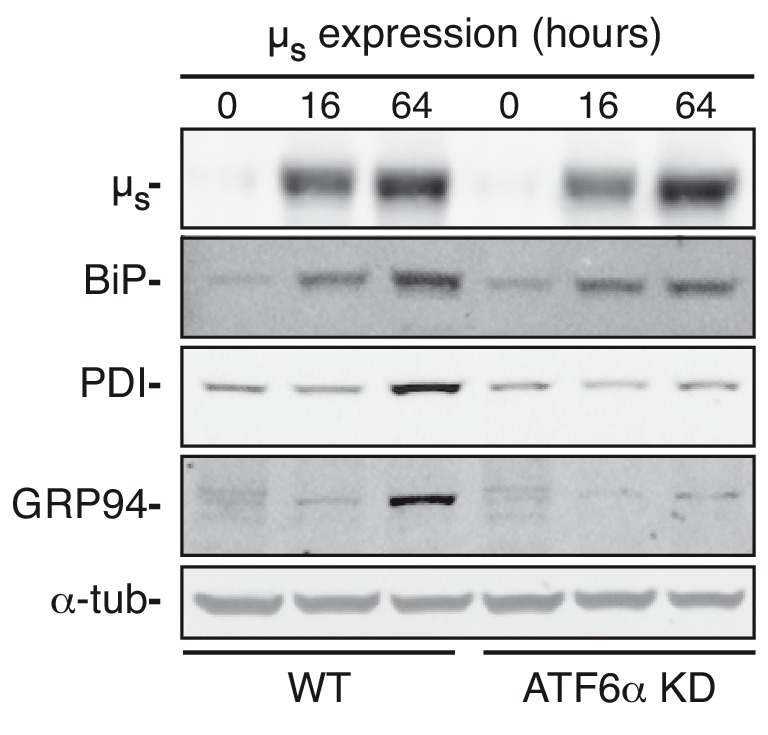
ATF6α ablation compromises ER chaperone upregulation upon µ_s_ expression. HeLa-µ_s_ cells in which ATF6α was silenced or not (WT), as indicated, were induced with 0.5 nM Mif to express µ_s_ for the indicated times. Levels of µ_s_, BiP, GRP94, PDI, and α-tubulin, were assessed by immunoblotting.

### ER stress and ensuing cytotoxicity levels correlate with the extent of µ_s_ being chaperoned

**Figure 3.**
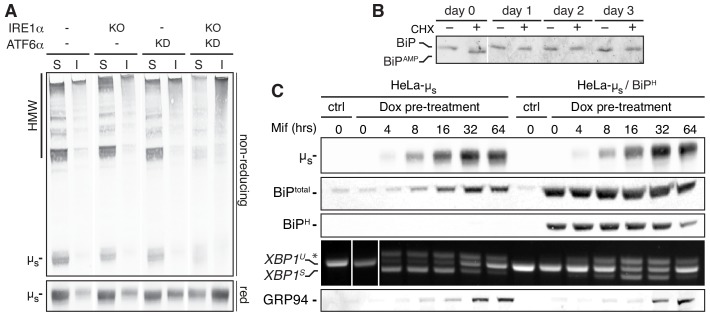
ER stress correlates with the extent of ER chaperones being engaged and becomes cytotoxic when their capacity is exceeded. (**A**) HeLa-µ_s_ cells, in which IRE1α (KO) and/or ATF6α (KD) was ablated, or not (-), as indicated, were induced with 0.5 nM Mif to express µ_s_ for 24 hr. Samples were lysed in NP40 and equivalent amounts of soluble (S) and insoluble (I) fractions resolved under reducing (red) or non-reducing conditions, blotted and decorated with anti-µ_s_. (**B**) HeLa-µ_s_ cells were induced with 0.5 nM Mif to express µ_s_ for the indicated times and treated with or without 100 μg/ml CHX for 3 hr before harvesting. Samples were analyzed by iso-electric focusing (IEF) to separate AMPylated (BiP^AMP^) from non-AMPylated BiP, which were detected by immunoblotting, as described (Preissler et al., 2015). To allow a better comparison between samples, considering the upregulation of BiP upon µ_s_ expression, approximately 15 µg of lysates were loaded for the 0 day samples, while only 2.5 µg were loaded for the other days. (**C**) HeLa-µ_s_-derived cells, harboring Dox-inducible hamster BiP (HeLa-µ_s_/BiP^H^), were treated for 2 days with 50 nM Dox to induce hamster BiP expression, while WT HeLa-µ_s_ cells were mock-treated with 50 nM Dox, before both cell lines were induced with 0.5 nM Mif to express µ_s_ for the indicated times. Immunoblotting of lysates revealed levels of µ_s_, total BiP, hamster BiP, and GRP94. *XBP1* mRNA splicing was assessed as in Figure 1C.

Since the UPR induces expression of ER resident chaperones, we surmised that µ_s_-driven ER stress becomes cytotoxic when the UPR is compromised, in particular upon ATF6α ablation, due to ‘under-chaperoning’ of µ_s_. Proteins that undergo folding tend to aggregate in absence of sufficient folding assistance. Upon ablation of IRE1α and ATF6α, µ_s_ indeed formed extensively disulfide-linked high molecular weight species that partitioned into a NP40-insoluble fraction, indicative of aggregation (Mattioli et al., 2006; Valetti et al., 1991)—with the single ablations showing intermediate phenotypes—(Figure 3A).

Under basal conditions, a significant proportion of BiP readily converts into an inactive, AMPylated state upon a three-hour block of protein synthesis with cycloheximide (CHX) (Figure 3B), which indicates that BiP gets to be dismissed from its chaperoning duties once its regular clients have had sufficient time to complete their folding, as has been reported before (Preissler et al., 2015). Conversely, in µ_s_-expressing cells no AMPylation occurred upon CHX treatment at any time upon the onset of µ_s_ expression (Figure 3B), suggesting that the vast majority of BiP is permanently engaged in chaperoning µ_s_ even though the BiP pool is expanding massively in response to µ_s_ expression (Bakunts et al., 2017).

As BiP stands out as a key chaperone for orphan µ_s_, we reasoned that the level of BiP at basal conditions is a key determinant for µ_s_-driven ER stress susceptibility. To test this idea, we created a derivative of the HeLa-µ_s_ cell line with an integrated copy of the hamster HSPA5 gene that encodes BiP under control of doxycycline (Dox). The induction of µ_s_ with Mif leads to it being the most abundantly transcribed gene (Bakunts et al., 2017) in the cells and concomitant induction of other transgenes would lead to competition for the transcription and/or translation machineries (not shown), thereby mitigating µ_s_ expression and, hence, µ_s_-driven ER stress by default. We therefore decided to pre-emptively enhance BiP levels with Dox at least ~10 fold prior to induction of µ_s_ expression (Figure 3C). Even though exogenously driven BiP transcription ceased after that, exogenous (hamster) BiP levels remained high for a prolonged time (Figure 3C).

In line with the notion that ER stress sensing in the HeLa-µ_s_ model occurs in a µ_s_/BiP ratiometric fashion (Bakunts et al., 2017), and in line with earlier reports that BiP overexpression dampens UPR activation (Bertolotti et al., 2000), *XBP1* mRNA splicing and upregulation of the UPR target GRP94 occurred with a delay when BiP levels were exogenously boosted as compared to when BiP was at endogenous levels, in spite of the similar extent and kinetics of µ_s_ accumulation (Figure 3C). Altogether the HeLa-µ_s_ model thus provides further support that sensing of ER stress correlates with the extent of the folding machinery being engaged in chaperoning its clients, and that BiP sequestration by client proteins appears to serve as the main proxy for that.

### Turnover of µ_s_ as afforded by ERAD is remarkably robust

**Figure 4.**
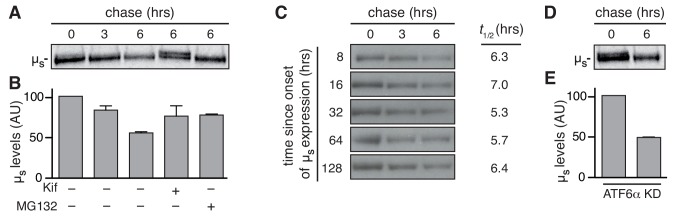
ERAD accounts for disposal of µ_s_ in a robust manner. HeLa-µ_s_ cells, in which ATF6α was ablated (**D,E**), as indicated, or not (**A-C**) were pulse labeled for 10 min and chased with excess unlabeled cysteine and methionine for the indicated times after 24 hr (**A,B,D,E**) or at various times (**C**), as indicated, after induction of µ_s_ expression with 0.5 nM Mif, in the absence (**A,C,D**) or presence (**A**) of 10 µM MG132 or 30 µM Kif, as indicated (+). Signals were quantitated and the signal after 0 hr chase was set at 100; mean and s.e.m. are shown in bar graphs (**B,E**); linear fitting of the quantitations of (**C**) were used to calculate the *t_½_* of µ_s_ at various time points after induction of its expression; see column on the right of panel. 10.7554/eLife.41168.012Figure 4—source data 1.

While µ_s_ levels increase, and the ER expands (~3–4 fold compared to basal levels), as wild-type cells are still adapting to the proteostatic insult, there is no further build-up of µ_s_ levels and ER expansion after ~2–3 days once homeostasis is restored (Bakunts et al., 2017), which implies that at that stage the influx of µ_s_ molecules into the ER must be matched by countermeasures. Translational attenuation through PERK activation can alleviate the burden on the ER folding machinery by diminishing the input of nascent clients entering the ER lumen (Harding et al., 1999). Yet, we ruled out that PERK-driven translational attenuation was a key determinant for ER homeostatic readjustment in the HeLa-µ_s_ model, considering that PERK ablation hardly impeded cell growth upon µ_s_ expression (Figure 1A,B). Accordingly, there was only a marginal reduction in overall protein synthesis (being at the lowest ~80% of that before induction) that was moreover transient (i.e. only manifest during the first 16 hr of µ_s_-expression) (Figure 4—figure supplement 1). Following the same reasoning, we also ruled out that regulated IRE1α-dependent decay (RIDD) (Hollien and Weissman, 2006; Hollien et al., 2009) of mRNAs that encode ER client proteins (and thereby limiting their influx into the ER) is important for homeostatic readjustment upon µ_s_ expression, since ablation of IRE1α had negligible impact on cell growth (Figure 1A,B). However, µ_s_ is a target of ERAD, as has been shown in plasma cells (Fagioli and Sitia, 2001), and which is shown here for the HeLa-µ_s_ cell model, since the proteasomal inhibitor MG132 to a large extent stabilizes µ_s_ levels in pulse-chase assays (Figure 4A,B). Since µ_s_ is glycosylated, it is subject to mannose trimming (Aebi et al., 2010), which is a key step in delivering µ_s_ to the retro-translocation machinery that shuttles it to the cytosol for proteasomal degradation (Fagioli and Sitia, 2001). Accordingly, the ER mannosidase I inhibitor kifunensine (Kif) stabilized µ_s_ in a similar manner as MG132 (Figure 4A,B).

**Figure 4—figure supplement 1.**
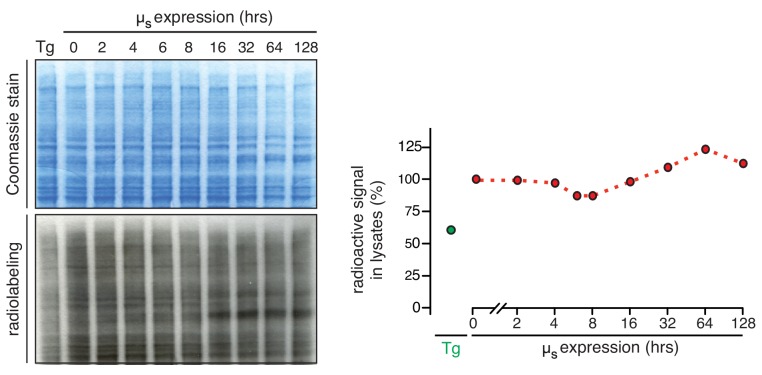
Translation is transiently attenuated upon µ_s_ overexpression to a marginal extent. HeLa-µ_s_ cells were induced with 0.5 nM Mif to express µ_s_ for the indicated times, or treated with 4 µM Tg for 45 min before they were pulse labeled for 10 min with ^35^S labeled methionine and cysteine for 10 min at the indicated times after induction. Lysates were separated by gel electrophoresis and total proteins were visualized by Coomassie, while radio-labeled proteins were detected and quantified by phosphor imaging. Labeling efficiency was compared to that in cells before induction, which was set at 100%. 10.7554/eLife.41168.010Figure 4—figure supplement 1—source data 1.

**Figure 4—figure supplement 2.**
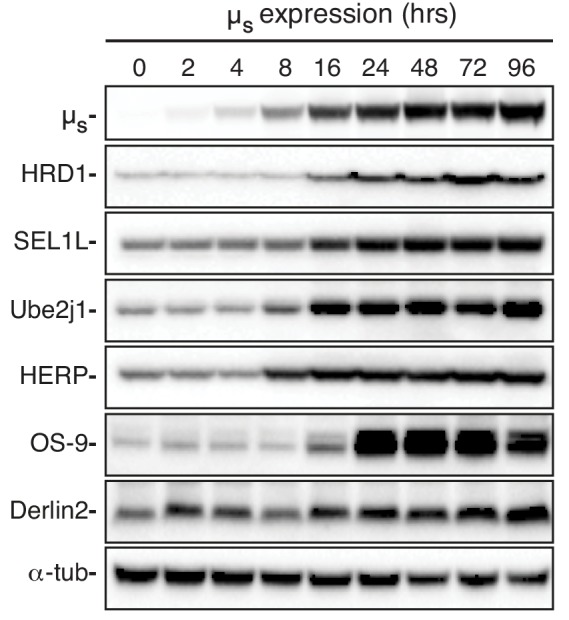
Levels of various ERAD components are induced in response to µ_s_ expression. HeLa-µ_s_ cells were induced with 0.5 nM Mif to express µ_s_ for the indicated times. Levels of µ_s_, HRD1, SEL1L, Ube2j1, HERP, Derlin-2, OS-9, and α–Tubulin were assessed by immunoblotting.

Interestingly, while µ_s_ levels built up steadily in the ER with time, ERAD kinetics hardly changed (i.e. the half-life (*t_½_*) of µ_s_ was remarkably constant), which implies that ERAD prowess kept pace with the accumulating load of µ_s_ (Figure 4C). ERAD components are UPR target genes (Walter and Ron, 2011), and indeed various major ERAD components (HRD1, SEL1L, Ube2j1, HERP, and OS-9), which we previously failed to detect by proteomics (Bakunts et al., 2017), were upregulated upon µ_s_ expression (Figure 4—figure supplement 2), Yet, their upregulation apparently serves at most to maintain rather than to reinvigorate ERAD kinetics of the accumulating µ_s_ load. In fact, ERAD kinetics of µ_s_ were not markedly affected by ablation of ATF6α (Figure 4D,E), in line with the finding that intracellular µ_s_ accumulation was not aggravated upon ATF6α ablation (Figure 2C,D). Thus, ER homeostatic failure upon ATF6α ablation is not due to compromised ERAD. Apparently, the upkeep of ERAD is robust in HeLa-µ_s_ cells, since we can also rule out that IRE1α and/or PERK are essential for maintaining sufficient ERAD capacity, as their ablation hardly caused any growth impairment of µ_s_-expressing cells (Figure 1A,B), unlike when ERAD is inhibited—see below.

### Disposal of µ_s_ through HRD1 complex-mediated ERAD is key for homeostatic readjustment

**Figure 5.**
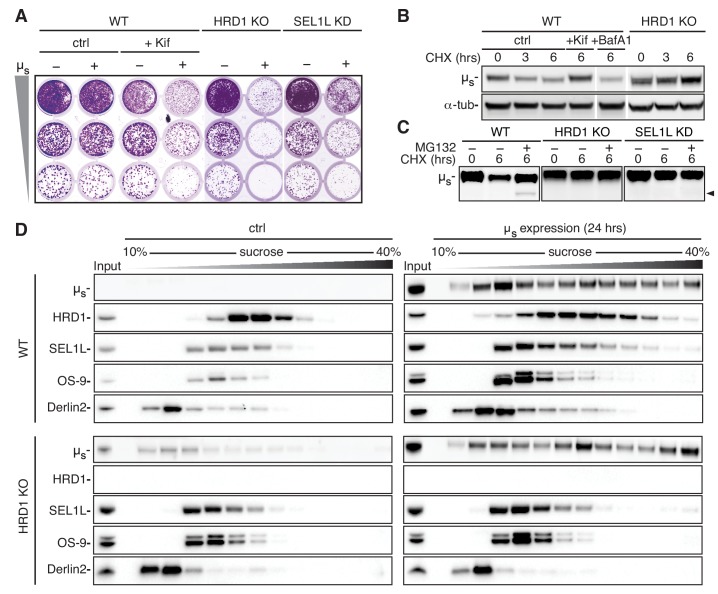
ERAD of µ_s_ is mediated through the HRD1 complex. (**A**) Growth assay as in Figure 1A of HeLa-µ_s_ cells, in which HRD1 was deleted (KO), SEL1L was silenced (KD), or not (WT). Cells were treated with 0.5 nM mifepristone (Mif) to induce expression of µ_s_ (+), or not (-), and WT cells were treated with Kif or not (ctrl), as indicated. (**B,C**) Immunoblots of µ_s_ harvested from WT, HRD1 KO (**B,C**), or SEL1L KD (**C**) HeLa-µ_s_ cells that were induced with 0.5 nM Mif to express µ_s_ for 4 hr and then treated for the indicated times with 100 μg/ml CHX either alone (**B,C**), in combination with 20 mM Kif, 100 nM BafA1, or not (ctrl) (**B**), or 10 µg/ml MG132 (**C**), as indicated. The arrowhead indicates the deglycosylated form of µ_s_. (**D**) HeLa-µ_s_ WT or HRD1 KO cells were induced with Mif (0.5 nM) for 24 hr to express µ_s_ or not (ctrl) , as indicated. Samples were lysed in 1% lauryl maltose neopentyl glycol (LMNG) and sedimented over a 10–40% sucrose gradient. Levels of µ_s_, HRD1, SEL1L, Derlin-2, and OS-9 were detected by immunoblotting. Note that in HRD1 KO cells leaky expression of µ_s_ becomes apparent due to the lack of ERAD. At low expression levels, however, µ_s_ does not form high molecular weight aggregates, indicative of the adequacy of the chaperoning machinery.

While prolonged proteasomal inhibition in itself is cytotoxic, blocking ERAD of glycoproteins with Kif per se did not affect cell viability (Figure 5A), and did not activate the UPR either (Figure 5—figure supplement 1). We reasoned that ERAD would be important, however, to hold bulk accumulation of µ_s_ in check. Indeed, viability was compromised in Kif-treated µ_s_-expressing cells (Figure 5A). Key ERAD components are the E3 ligase HRD1 and its partner SEL1L (Olzmann et al., 2013), which have previously been shown to mediate ERAD of µ_s_ (Cattaneo et al., 2008). Indeed, ablation of HRD1 and, to a lesser extent, of SEL1L was synthetically lethal in HeLa-µ_s_ cells upon µ_s_ expression (Figure 5A).

**Figure 5—figure supplement 1.**
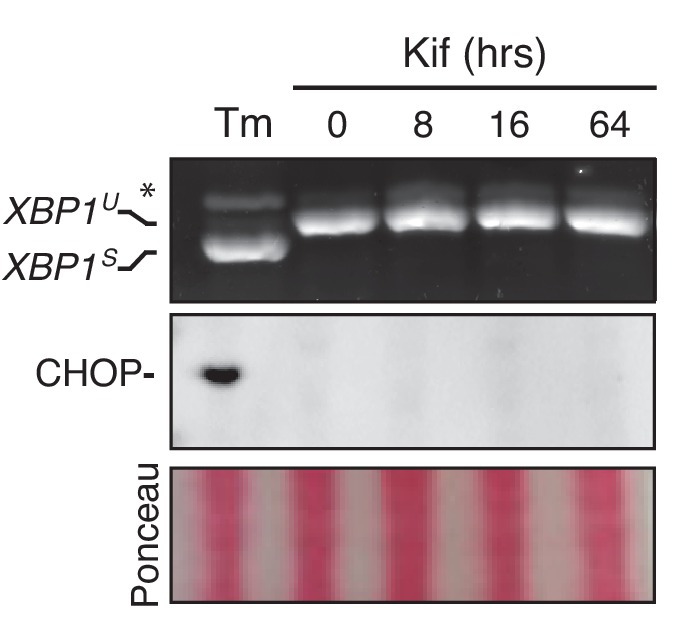
Inhibition of ERAD does not trigger the UPR under basal conditions. HeLa-µ_s_ cells were treated with 30 µM Kif for the indicated times, or treated with 5 µg/ml Tm for 4 hr as a positive reference. *XBP1* mRNA splicing and CHOP levels were assessed as in Figure 1C. Ponceau staining of the blot served as a loading control.

**Figure 5—figure supplement 2 .**
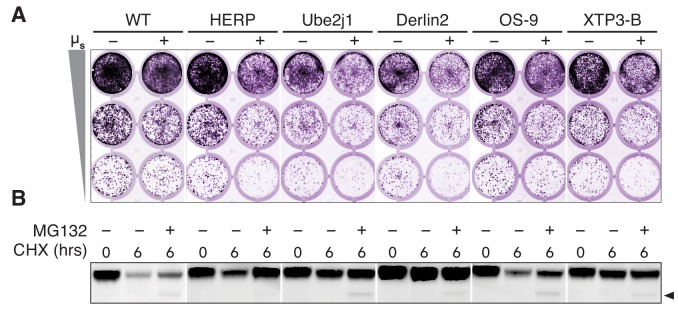
HERP, Ube2j1, and Derlin-2 are key for ERAD of µ_s_. (**A**) Growth assays as in Figure 1A of WT HeLa-µ_s_ cells, or in which Ube2j1, HERP, Derlin-2, OS-9, or XTP3-B were deleted by CRISPR/Cas9 in a non-clonal fashion (KO). (**B**) Immunoblot of µ_s_ harvested from these same cells that were induced with 0.5 nM Mif to express µs for 4 hrs and then treated (or not) for 6 hrs with 100 μg/ml CHX either alone or in combination with 10 µg/ml MG132, as indicated. The arrowhead marks deglycosylated µ_s_.

HRD1 and SEL1L cooperate to target ERAD substrates back across the ER membrane to the cytosol, where substrates are ubiquitinated, deglycosylated by N-glycanase, and, ultimately, degraded by the proteasome (Olzmann et al., 2013). Accordingly, µ_s_ was stabilized in HRD1 KO or SEL1L KD cells, similarly as upon Kif treatment of WT cells, while in ERAD-competent WT cells µ_s_ was degraded upon CHX treatment (Figure 5B,C). Proteasomal inhibition with MG132 stabilized µ_s_ in WT cells, and the appearance of a deglycosylated form of µ_s_ confirmed that, at least of fraction of µ_s_ was retrotranslocated to the cytosol, and accessible to N-glycanase. Interestingly, in HRD1 KO or SEL1L KD cells no deglycosylated form of µ_s_ appeared, indicating that disposal of µ_s_ was blocked at (or prior to) the retrotranslocation step (Figure 5C).

There appear to be more than 25 other E3 ligases that localize at the ER membrane next to HRD1 (Neutzner et al., 2011; Kaneko et al., 2016), but, curiously, none of these can compensate for the loss of HRD1. Furthermore, treatment with the autophagy inhibitor Bafilomycin A1 (BafA1) did not lead to any stabilization of µ_s_ (Figure 5B). Thus, HRD1-mediated ERAD is the main if not exclusive disposal mechanism that is essential for ER homeostatic readjustment in the HeLa-µ_s_ model, even though autophagy has been reported to curtail IgM production and ER expansion in plasma cells (Pengo et al., 2013).

The synthetic lethality that ensues once ERAD is compromised in the HeLa-µ_s_ model offered a powerful tool to define which factors are crucial to act in conjunction with HRD1 and SEL1L in the disposal of µ_s_. To that end, we ablated several candidate HRD1 partners by CRISPR/Cas9 (but without clonal selection; that is without necessarily reaching fully penetrant phenotypes). In this initial survey, we witnessed that cell viability upon µ_s_-expression was compromised, and that µ_s_ was stabilized in a CHX chase by ablation of HERP, Ube2j1, and Derlin2 to significant extents (Figure 5—figure supplement 2). However, ablation of OS-9 or of XTP3-B only mildly affected µ_s_-expressing cells. These two lectins indeed have been shown previously to be interchangeable, as they capture soluble ERAD substrates upon mannose trimming of their glycans before handing over these substrates to SEL1L (Bernasconi et al., 2010; van der Goot et al., 2018).

In sedimentation gradients HRD1, SEL1L, and Derlin2 shifted towards heavier fractions upon µ_s_ expression, while the redundant ERAD factor OS-9 did not (Figure 5D). These findings indicate that disposal of µ_s_ is effectuated through assembly of higher-order ERAD-mediating complexes with, at the least, HRD1, SEL1L, and Derlin2 at their core. These complexes nucleate around HRD1 as its ablation abrogated their formation (Figure 5D).

### Homeostatic failure upon ERAD inhibition coincides with µ_s_ levels outpacing BiP upregulation

**Figure 6.**
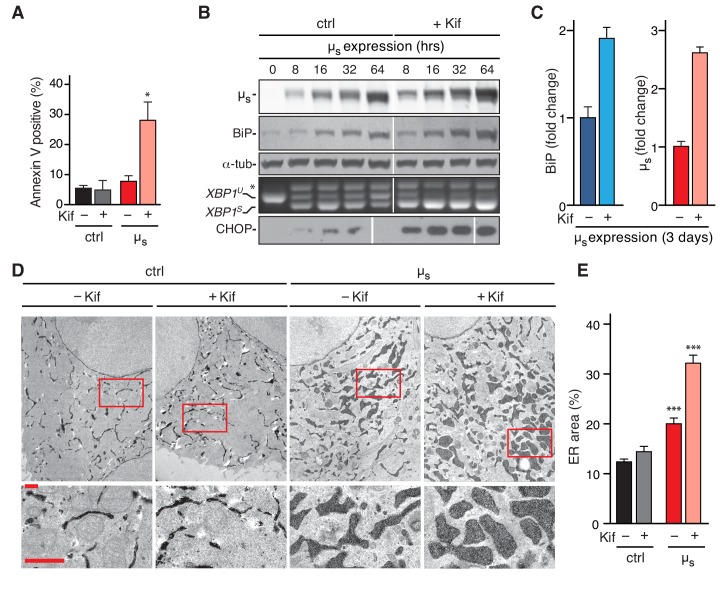
Abrogation of µ_s_ disposal through ERAD leads to BiP being permanently eclipsed, ER homeostatic failure, and apoptosis. (**A,D,E**) HeLa-µ_s_ cells, harboring APEX-KDEL (**D,E**) or not (**A**) were induced with (µ_s_) or without (ctrl) 0.5 nM Mif for 3 days in the presence or absence of 30 µM Kif. (**A**) Percentages of Annexin V positive cells were assessed by cytometric analysis. Mean and s.e.m. are shown in a bar graph, n = 2–4. (**B,C**) HeLa-µ_s_ cells were induced to express µ_s_ for various times as indicated (**B**) or for 3 days in the absence or presence of 30 µM Kif. (**C**) Levels of µ_s_, BiP, and α-tubulin as well as activation of the IRE1α and PERK branches of the UPR were assessed as in (Bakunts et al., 2017). (**B**) Levels of BiP and µ_s_ were assessed by quantitative immunoblotting as described (Bakunts et al., 2017), and depicted in bar graphs as in Figure 2D,E, such that the µ_s_ levels in the absence of Kif were scaled to BiP levels at a ratio of 2:3. Levels in the presence of Kif are expressed as a fold change compared to levels in the absence of Kif; mean and s.e.m. are shown; n = 2. (**D**) In cells harboring APEX-KDEL the extent of ER expansion was assessed as in Figure 2A. Boxed areas are shown by 3-fold magnification; scale bars represent 1 µm. The percentage of the dark area within the cytoplasm corresponding to ER was determined and depicted in bar graphs (**E**), mean and s.e.m. are shown, n = 10. Statistical significance of differences in Annexin V staining (**A**), or the extent of ER occupying cytosolic area in the electron micrographs (**E**) were tested by ANOVA (*p≤0.05; ***p≤0.001). 10.7554/eLife.41168.017Figure 6—source data 1.

When ERAD is functional, an excess of BiP over µ_s_ is restored upon 3 days of µ_s_ expression. The BiP:µ_s_ stoichiometry is then ~3:2, as estimated from a combination of quantitative immunoblotting and proteomics techniques (Bakunts et al., 2017). As soon as BiP levels are in excess again, UPR signaling subsides to submaximal output, and ER homeostatic readjustment to µ_s_ expression is successful (Bakunts et al., 2017). The loss of viability in Kif-treated µ_s_-expressing cells (Figure 5A) indicated that ER homeostatic readjustment failed, and these cells indeed underwent apoptosis (Figure 6A). Homeostatic failure in Kif-treated µ_s_-expressing cells entailed that ER stress was unresolved, and accordingly, IRE1α and PERK chronically signaled at maximal levels (Figure 6B).

Chronic maximal UPR activation upon ERAD inhibition in µ_s_-expressing cells implied that induction of BiP expression was persistently at maximal levels. Nevertheless, the build-up of BiP levels (~2 fold further increase after 3 days), could not keep pace with the augmented accumulation of µ_s_ (~3 fold further increase after 3 days) upon ERAD inhibition, such that µ_s_ reached levels in the ER that were at about a 1:1 stoichiometry with BiP (Figure 6C). Indeed, aggregation of µ_s_ increased when ERAD was defective, as judged by µ_s_ shifting more towards heavier fractions in HRD1 KO than in WT cells (Figure 5D). Thus, under those conditions the chaperoning machinery becomes limiting, similarly as upon ablation of IRE1α and/or ATF6α in ERAD-competent cells (Figure 3A).

We previously estimated the volume of the ER under basal conditions to be (0.10–0.12)^3/2^≈3–4% of the cytoplasmic volume, and upon 3 days of µ_s_ expression to be (0.18–0.20)^3/2^≈7–8% of the cytoplasmic volume, corresponding to a ~ 2–3 fold increase of ER volume (Bakunts et al., 2017). Upon ERAD inhibition with Kif the ER did not markedly expand in non-µ_s_-expressing cells. In µ_s_-expressing cells, instead, ERAD inhibition caused the area of ER staining within the cytoplasm to reach 30–35%, which on a rough estimate would account for (0.3–0.35)^3/2^≈17–20% of the cytoplasmic volume, implying that the ER had expanded ~6–7 fold since the onset of µ_s_-expression (Figure 6D,E).

We concluded that curtailing the µ_s_ load by ERAD is essential for the cells to cope with µ_s_ expression in bulk. ER homeostatic failure upon ERAD inhibition coincided with an inadequacy to raise BiP levels in sufficient excess over those of µ_s_ and, hence, with its ‘under-chaperoning’, in spite of the impressive BiP upregulation and ER expansion at large. Thus, in absence of ERAD, not only the chronic maximal UPR activation, but also the µ_s_–driven proteotoxicity appear to be due to BiP running short, similarly as when UPR signaling was compromised upon ablation of ATF6α (Figure 1C,D; Figure 2C–E).

### Sequestration of BiP is both necessary and sufficient for UPR activation and ER stress-provoked proteotoxicity

**Figure 7.**
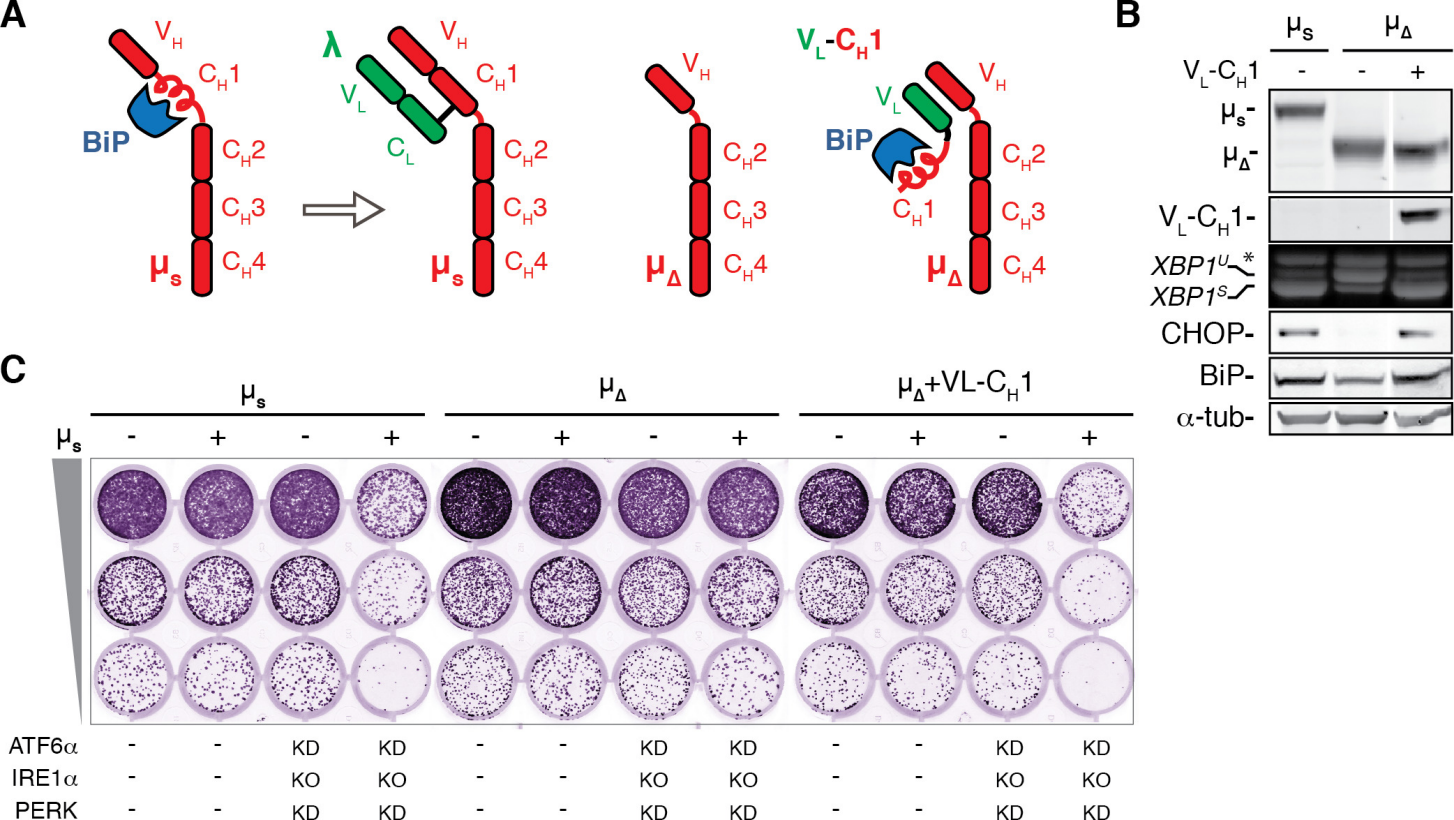
The BiP-sequestering C_H_1 domain of µ_s_ is necessary and sufficient to cause UPR activation and proteotoxic ER stress in absence of the UPR. (**A**) Schematic representation of BiP associating with the C_H_1 domain of µ_s_ until it is displaced by the light chain (λ). Deletion of the C_H_1 domain (µ_∆_) abolishes BiP association, but through pairing of the V_H_ and V_L_ domains, the C_H_1 domain can associate in trans by virtue of a synthetic chimeric V_L_-C_H_1 construct. (**B**) HeLa cells were induced for 24 hr with 0.5 nM Mif to express the transgenes µ_s_, µ_s_∆C_H_1 (µ_∆_) alone or in conjunction with V_L_-C_H_1, as indicated. Immunoblotting of lysates revealed levels of µ_s_, µ_∆_, V_L_-C_H_1, BiP, CHOP, and α-tubulin, as in Figure 1A. (**C**) Growth assay as in Figure 1A of HeLa cells inducibly expressing µ_s_, µ_s_∆C_H_1 (µ_∆_) in conjunction with V_L_-C_H_1 or not, and in which the UPR was ablated (i.e. IRE1α was deleted (KO), and ATF6α and PERK were silenced in combination), or not, as indicated.

In plasma cells BiP stringently interacts with µ_s_ through the C_H_1 domain, until it is displaced by the light chain (Bole et al., 1986; Figure 7A), which makes µ_s_ an unusual ER client. Evolutionary pressure against secretion of orphan µ_s_ (i.e. unaccompanied by the light chain) must have been extraordinarily high for obvious immunological reasons (Anelli and van Anken, 2013), which would explain the exceptionally strong affinity of the C_H_1 domain for BiP, that is to let BiP mediate stringent ER retention of unpaired µ_s_. Thus, we reasoned that removal of the BiP binding C_H_1 domain from µ_s_ (Figure 7A), would offer an ideal tool to validate whether limitations in BiP availability define both the amplitude of UPR activation as well as any proteotoxicity that would ensue from overexpression of ER client proteins. In line with our model, µ_s_∆C_H_1 hardly activated the UPR, as shown for the IRE1α and PERK branches, despite being expressed at similar levels as µ_s_ wild-type (Figure 7B). Moreover, genetic ablation of the three UPR pathways failed to cause synthetic lethality in µ_s_∆C_H_1-expressing cells (Figure 7C). Conversely, co-expression of µ_s_∆C_H_1 with a chimeric protein consisting of the variable domain of λ fused with the C_H_1 domain of µ_s_ (V_L_-C_H_1), which teams up with µ_s_∆C_H_1 through interactions between V_L_ with the variable domain of µ_s_ (V_H_) (Figure 7A), restored UPR activation (Figure 7B) and synthetic lethality upon UPR ablation (Figure 7C). These findings corroborate that the BiP sequestering C_H_1 domain of µ_s_ causes UPR activation, as well as proteotoxicity when reinforcement of BiP levels through the UPR is inadequate.

## Discussion

The fact that BiP plays a key role in regulating the UPR has been known for almost 20 years (Bertolotti et al., 2000). Overexpression of BiP dampens UPR activation (Bertolotti et al., 2000), which we confirm with the data presented here, while inactivating BiP with the AB5 subtilase cytotoxin acutely causes ER stress and UPR activation (Paton et al., 2006). Yet, by employment of a proteostatic stimulus with a single well-defined BiP binding module as the source of ER stress, we have provided here experimental evidence that defines both proteostatic ER stress, and the resulting activation of the UPR, to be the specific consequence of insufficient BiP availability. Both the UPR and ERAD redress the relative BiP shortage, and thus counteract that the proteostatic stress becomes proteotoxic. BiP indeed has been acclaimed as the master regulator of ER function (Hendershot, 2004), and various cytotoxic consequences may follow from the excessive sequestering of BiP by µ_s_ that precludes BiP from attending to its other functions. For instance, BiP closes off the translocon, and efflux of Ca^2+^ from the ER into the cytosol through poorly gated translocons may already be sufficient to cause apoptosis (Schäuble et al., 2012).

Our data emphasize that proteotoxicity stemming from the accumulation of client proteins in the ER is not the result of UPR signaling, as often is assumed from the notion that the UPR can initiate pro-apoptotic pathways. Instead, the UPR foremost counteracts proteotoxicity by inducing the ER resident folding machinery (most in particular BiP). In light of our data, the capacity of the UPR to switch from cytoprotective to pro-apoptotic signaling may well have arisen in metazoans to pre-emptively eliminate cells in which restoration of ER homeostasis is unachievable, and, hence, cell death has become inevitable.

Perhaps surprisingly, our results furthermore highlight that PERK and IRE1α are dispensable for successful ER homeostatic readjustment to the µ_s_ stimulus in HeLa cells. Apparently, the PERK-mediated translational block, which is only transient, offers negligible advantage when cells face a sudden proteostatic insult that sequesters BiP (and/or the ER chaperone machinery at large) in a persistent manner. PERK-mediated translational attenuation instead may be required in particular to sustain episodic secretory activity, such as in β-cells of the pancreas. PERK KO mice indeed suffer mostly from degeneration of tissues with episodic secretory activity (Zhang et al., 2002). Similarly, requirements for the IRE1α/XBP1 pathway seem to be tissue-specific. Both deletion of IRE1α (Urano et al., 2000; Zhang et al., 2005) and of XBP1 (Reimold et al., 2000) cause embryonic lethality, but XBP1 KO mice are rescued with an XBP1 transgene specifically expressed in the liver (Lee et al., 2005), while IRE1α KO mice are rescued when the placenta expresses IRE1α (Iwawaki et al., 2009), and the resulting rescued mice display relatively mild symptoms, that ishyperglycemia, hypoinsulinemia, and decreased antibody titers, despite the lack of IRE1α (Iwawaki et al., 2010). In line with our findings, homeostatic readjustment to ER stress in most mammalian tissues seems to rely mainly on ATF6 proteins (Wu et al., 2007; Yamamoto et al., 2007), that is ATF6α, and its related ER stress sensor ATF6β. The ATF6α/β double KO confers embryonic lethality (Yamamoto et al., 2007). At present, it is unclear whether embryonic lethality of the ATF6α/β double KO can be rescued, for instance through enhancement of other UPR branches.

Finally, since proteotoxicity due to the accumulation of (mutant) proteins in the ER seems to play a key role in various types of disease (Anelli and Sitia, 2010; Cao and Kaufman, 2014), our insights may be of relevance for the design of drugs aimed at alleviating ER stress (Hetz and Papa, 2018), and hence proteotoxicity stemming from ER stress. We argue that pharmacological intervention against pathogenic ER stress foremost should promote a favorable ratio of BiP levels over those of its disease-causing client protein.

## Materials and methods

All assays were performed as described (Bakunts et al., 2017), except that in addition, along the same principles as described (Bakunts et al., 2017), the following cell lines were derived by clonal selection from either HeLa-µ_s_ or HeLa-MifON, as summarized in Supplementary file 1: HeLa-µ_s_ HRD1 KO, and HeLa-µ_s_-BiP^H^, which inducibly (by Dox) expresses hamster BiP, HeLa-µ_s_∆C_H_1, which inducibly (by Mif) expresses µ_s_∆C_H_1, and HeLa-µ_s_∆C_H_1/V_L_-C_H_1, which inducibly (by Mif) expresses µ_s_∆C_H_1 in combination with V_L_-C_H_1. At least three independent clones of HeLa-µ_s_ HRD1 KO cells were tested in phenotypic assays to rule out off-target effects. CRISPR/Cas9-mediated depletion of HERP, Ube2j1, Derlin-2, OS-9, or XTP3-B in HeLa-µ_s_ was performed using single guide RNA (sgRNA) sequences (Supplementary file 1) cloned into the PX459 vector (Addgene #62988) and were used as puromycin-selected pools without clonal isolation. Cloning into PX459 was performed as described previously (Ran et al., 2013). sgRNA target sequences for Hrd1, HERP (Schulz et al., 2017), Derlin-2, Ube2j1 (Ma et al., 2015), OS-9, and XTP3-B (van der Goot et al., 2018) have been described previously. Silencing of SEL1L was obtained using ON-Target SMARTpool siRNA from Dharmacon.

The inducible hamster BiP, µ_s_∆C_H_1 and V_L_-C_H_1 cassettes were created by standard molecular biology techniques from the cDNAs described in Bakunts et al. (2017). The C_H_1 domain (E140-P244) was deleted from µ_s_ in µ_s_∆C_H_1. That same C_H_1 domain was placed downstream of V127 of λ, replacing the C_L_ domain, to create the chimeric V_L_-C_H_1 construct. A myc tag (EQKLISEEDL) was placed at the C-terminus of V_L_-C_H_1 for immunodetection purposes. Cells were routinely tested, that is on a monthly basis, to be mycoplasm-free by use of a standard diagnostic PCR. All cell lines in this study were ultimately derived from HeLa S3 cells, of which the genotype was confirmed by PCR single locus technology. Antibodies used in addition to those described before (Bakunts et al., 2017) are summarized in Supplementary file 1.

To separate NP-40 soluble from insoluble fractions, cells were washed and lysed in 0.2% NP-40, 50 mM Tris-HCl pH 7.5, 150 mM NaCl, 5 mM EDTA, 10 mM *N-*ethylmaleimide and a cocktail of protease inhibitors. The NP-40-insoluble fractions were separated from the soluble fractions by centrifugation at 3,400 g for 10 min and the insoluble pellets were solubilized in 1% SDS, 50 mM Tris-HCl pH 7.5, 10 mM NEM for 10 min at RT and sonicated on ice. For fractionation of ERAD complexes cells were lysed in 1% lauryl maltose neopentyl glycol (LMNG, Anatrace) containing buffer (50 mM Tris-HCl, pH7.4, 150 mM NaCl, 5 mM EDTA) and lysates were loaded onto 10–40% sucrose gradients also containing 1% LMNG, formed by following the manufacturers’ instructions (Gradient Master, Biocomp). Sedimentation was achieved by centrifugation in a SW.41 swing bucket rotor (Beckman) at 39,000 rpm for 16 hr at 4°C. Thirteen fractions were collected from the top and proteins precipitated with TCA (trichloroacetic acid). Protein pellets were resuspended in Laemmli buffer containing DTT (10 mM), heated alongside 25 μg of the original lysates as input, at 56°C prior to separation by SDS-PAGE.

## Data Availability

All data generated or analysed during this study are included in the manuscript and supporting files.

## Additional files

Supplementary file 1. List of cell lines and reagents.

Transparent reporting form
